# Changing Trends in Firework-Related Eye Injuries in Southern China: A 5-Year Retrospective Study of 468 Cases

**DOI:** 10.1155/2020/6194519

**Published:** 2020-08-06

**Authors:** Fangyu Wang, Bingsheng Lou, Zhaoxin Jiang, Yao Yang, Xinqi Ma, Xiaofeng Lin

**Affiliations:** State Key Laboratory of Ophthalmology, Zhongshan Ophthalmic Center, Sun Yat-Sen University, Guangzhou 510060, China

## Abstract

Firework-related eye injury is a horrible medical problem and creates huge health and social burdens. Herein, we explored the changing trends and demographic and clinical features of firework-related eye injury, in an effort to inform strategies to prevent this injury. We reviewed the data of 468 hospitalized patients who underwent surgery for firework-related eye injury at the Zhongshan Ophthalmic Center between January 2013 and December 2017. During this period, the trend in firework-related eye injuries was stable (mean, 93 ± 14 cases per year), and fireworks were the major cause of explosive eye injury. The average age of the patients was 24 ± 18 years and 87% of the patients were male, with boys under 10 years of age comprising the largest group (27% of patients). There were an average of 24 ± 7 cases per year from urban areas and 70 ± 8 cases from rural areas (*P* < 0.05). Furthermore, 21 ± 5% of cases occurred during Spring Festival. After treatment, the best corrected visual acuity was increased compared to that before treatment, and the intraocular pressure tended to become normal by the final visit (*P* < 0.05). The top three diagnoses were cataract (39%), retinal detachment (18%), and choroidal detachment (14%). Additionally, the most common surgery was cataract extraction (25%), followed by pars plana vitrectomy (19%) and pars plana vitrectomy plus pars plana lensectomy (10%). Over the five-year study period, day surgery hospitalization increased from 1% to 32%. This was associated with a corresponding decrease in the length of hospitalization, without adverse events, demonstrating that day surgery is feasible in firework-related eye injury cases. The present study results suggest that greater attention should be paid to firework-related eye injury, and a variety of measures should be taken to prevent this kind of ocular tragedy.

## 1. Introduction

Firework-related eye injury is one of the most common injuries caused by fireworks [1–3]. Carelessness and improper use of fireworks can cause these terrible accidents [4, 5]. Firework-related eye injuries lead to structural and functional damage of the eyes and, in some cases, cause a permanent loss in vision. Not only do firework-related eye injuries cause great harm to the patient, they also place a heavy burden on the family. Thus, firework-related eye injuries can create heavy medical, economic, and social burdens [6–9]. This public health issue deserves greater attention.

In addition to minor eye injuries, fireworks can cause various serious ocular injuries, including traumatic cataract, intraocular foreign body, and retinal detachment (RD) [10, 11]. Patients with such serious injuries require hospitalization for surgery. An injured eye must be repaired immediately to restore its structure. Regarding functional recovery, additional complex operations may be necessary, depending on the follow-up condition. In view of these disastrous consequences, a systematic analysis of serious firework-related eye injuries is necessary.

In developed countries, reviews of firework-related eye injuries have raised public awareness [1, 7, 10–15]. However, such a review, especially one focused on serious cases, has not been largely reported in China. Thus, the changing trends and detailed clinical features of firework-related eye injury remain to be explored. The present study collected data from hospitalized patients who underwent surgery for firework-related eye injury at the Zhongshan Ophthalmic Center from 2013 to 2017. The Zhongshan Ophthalmic Center is the biggest ophthalmic center in China and accepts patients mainly from southern China, but also from the whole country. We aimed to provide an overview of firework-related eye injuries in China and disseminate its valuable clinical and demographic features.

## 2. Materials and Methods

This study was approved by the Medical Ethics Committee of Sun Yat-sen University. We reviewed the clinical records of all patients who were hospitalized and underwent surgery for firework-related eye injury at the Zhongshan Ophthalmic Center from January 2013 to December 2017. Data were provided by the medical record management department. Change over time in the numbers of explosive and firework-related eye injury cases was analyzed. Additionally, the various causes of explosive eye injury were evaluated as etiologic features, and age, sex, and regional distribution of patients were evaluated as demographic features. Furthermore, basic ocular data, including the best corrected visual acuity (BCVA) and intraocular pressure (IOP), main diagnosis on admission, type of surgery, and duration of hospitalization were analyzed.

SPSS version 13.0 (SPSS, Inc., Chicago, IL) was used for statistical analysis. Data are expressed as the mean ± standard deviation. The *t*-test was used to compare demographic data, and the Wilcoxon rank-signed test and Chi-square test were used to compare basic ocular data. Results were considered as statistically significant at *P* < 0.05.

## 3. Results

### 3.1. Number of Explosive and Firework-Related Eye Injury Cases and Major Causes of Explosive Eye Injury

**Figure 1:**
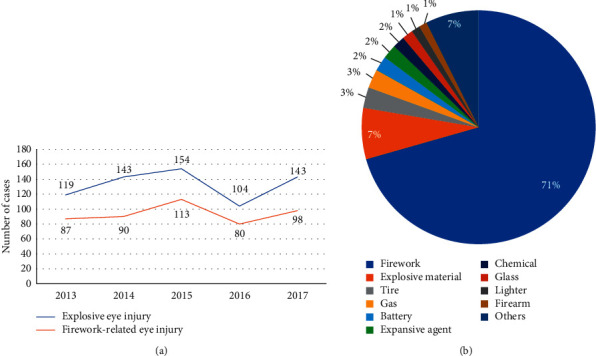
Number of explosive and firework-related eye injuries each year (a) and major causes of explosive eye injury (b). (a) There were an average of 133 ± 20 cases of explosive eye injury and 94 ± 13 cases of firework-related eye injury per year between 2013 and 2017. (b) Fireworks were the major cause (71%) of explosive eye injury, followed by explosive materials (7%) and tires (3%).

The numbers of explosive and firework-related eye injury cases are shown according to year in Figure 1(a). There were 663 cases of explosive eye injury during the 5-year study period, averaging 133 ± 20 cases per year. The number of cases was quite stable over time, with a slight decline in 2016. Furthermore, there were 468 cases of firework-related eye injury during the 5-year study period, averaging 94 ± 13 cases per year. The trend over time in firework-related eye injury followed the same pattern as that for explosive eye injury.

There were many causes of explosive eye injury (Figure 1(b)). Fireworks were the major cause of explosive eye injury (71%), followed by explosive materials (7%), tires (3%), and gas (3%).

### 3.2. Demographic Features of the Study Population

**Figure 2:**
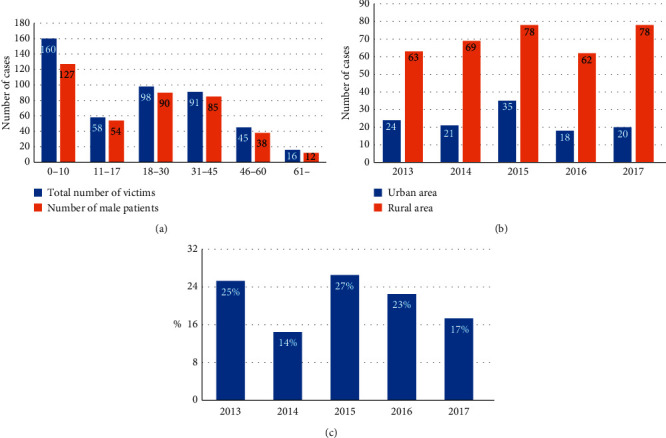
Age, sex, and regional distribution of patients (a, b) and proportion of firework-related eye injuries that occurred during spring festival (c). (a) The average age of the patients was 24 ± 18 years, 87% of patients were male, and 27% of patients were boys who were under 10 years old. (b) There was an average of 24 ± 7 patients from urban areas and 70 ± 8 patients from rural areas, per year between 2013 and 2017 (*P* < 0.05, *t*-test). (c) From 2013 to 2017, 21 ± 5% cases occurred during the Spring Festival, which was much higher than the average monthly incidence of 7.8%.

The age and sex distribution of patients with firework-related eye injuries are shown in Figure 2(a). The youngest patient was 7 months old, and the oldest was 86 years old. The mean age of the patients was 24 ± 18 years and 87% (406/468) of the patients were male. The largest group comprised boys who are under 10 years old, accounting for 27% (127/468) of patients.

The regional distribution of patients is shown in Figure 2(b). There was a yearly average of 24 ± 7 patients from urban areas and 70 ± 8 patients from rural areas, during the 5-year study period (*P* < 0.05).

The number of firework-related eye injury cases peaked at the time of Spring Festival (Figure 2(c)). During the 5-year study period, 21 ± 5% of cases occurred during Spring Festival, which was much higher than the average monthly incidence of 7.8%.

### 3.3. Ocular Clinical Features of Firework-Related Eye Injury

#### 3.3.1. Initial and Final BCVA and IOP

**Figure 3:**
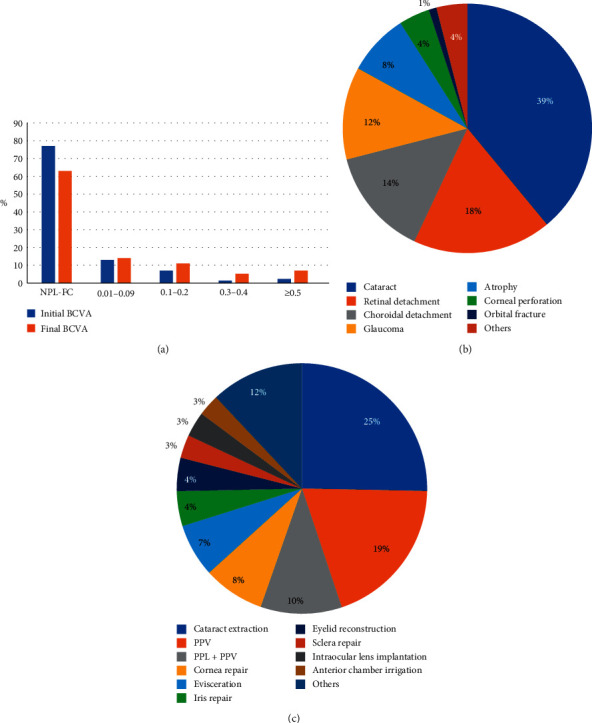
Ocular clinical features of patients with firework-related eye injury. (a) The BCVA in patients with firework-related eye injury, before and after treatment. (b) The diagnoses at admission are shown. (c) The major surgeries performed are shown. PPV, pars plana vitrectomy; PPL, pars plana lensectomy.

We analyzed the initial and final BCVA and IOP, with a mean follow-up interval of 9 months. Some injured eyes could not be examined and some patients could not complete the examination; thus, we analyzed the BCVA in 440 eyes (Figure 3(a)) and the IOP in 448 eyes (Table 1). The proportion of patients at the first level of no perception of light (NPL-FC) decreased from 77% at the initial visit to 63% at the final visit. For the remaining levels, the proportion of patients at the final visit exceeded the proportion of patients at the initial first visit. Thus, the BCVA was increased at the end of follow-up, relative to initial values (*P* < 0.05). Furthermore, the proportion of patients with an IOP within normal range increased from 55% at the initial visit to 69% at the final visit. Thus, the IOP tended to become normal by the final visit (*P* < 0.05).

#### 3.3.2. Main Clinical Diagnosis and Major Surgery Performed

The main diagnoses on admission are shown in Figure 3(b). The top three diagnoses were cataract (39%), RD (18%), and choroidal detachment (CD; 14%). Furthermore, 4% of patients had a combined injury of cataract, RD, and CD. The major surgeries performed are shown in Figure 3(c). The most common surgery was cataract extraction (25%), followed by pars plana vitrectomy (PPV; 19%), pars plana lensectomy plus PPV (PPL + PPV; 10%), cornea repair (8%), and evisceration (7%).

### 3.4. Hospitalization Days and Proportion of Day Surgeries

**Figure 4:**
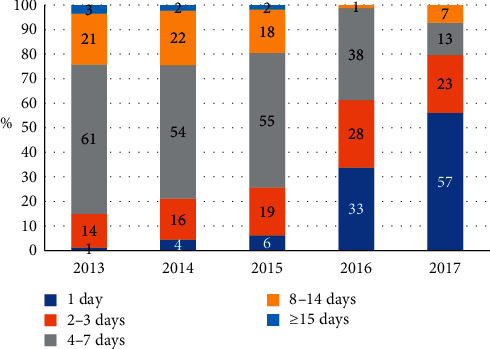
Hospitalization days in firework-related eye injury. In 2013 and 2014, few patients were hospitalized for only 1 day, and more than 50% of the patients stayed for 4–7 days. From 2015 to 2017, the proportion of patients who stayed for 1 day increased from 6% to 57%, while the proportion of patients who stayed for 4–7 days decreased from 55% to 13%.

Data regarding the number of hospitalization days are shown in Figure 4. In 2013 and 2014, few patients stayed for only 1 day, 15% of patients stayed for 2-3 days, more than 50% of patients stayed for 4–7 days, and 24% of patients stayed for more than 8 days. From 2015 to 2017, the proportion of patients who stayed for 1 day increased from 6% to 57%, about 23% of the patients stayed for 2-3 days, the proportion of patients who stayed for 4–7 days decreased from 55% to 13%, and the proportion patients who stayed for more than 8 days decreased from 20% to 7%.

## 4. Discussion

The present study showed that the number of firework-related eye injury cases was stable over a 5-year study period (2013 to 2017), with a slight decline in 2016. Additionally, fireworks were the major cause of explosive eye injury. Unterlauft et al. [10] reported on 149 firework-related eye injury cases in Germany, and Knox et al. [11] reported on 76 cases treated from 2004 to 2006 in the United Kingdom. However, data regarding changing trends in firework-related eye injuries are scarce. The number of cases likely remained stable in the present study because China did not ban fireworks everywhere, and the decline in 2016 may have resulted from new bans on fireworks imposed in large cities (e.g., Zhengzhou, Hangzhou, and Shanghai) in 2016. Thus, legislation is effective in reducing this kind of trauma [1, 10, 11, 16]. Furthermore, there are a few case reports on explosive eye injuries, such as colored corn starch dust and batteries [17, 18], but systematic reports regarding explosive eye injuries are lacking. Therefore, we analyzed the causes of explosive eye injury and found that fireworks were the major cause, indicating that we should pay more attention to this public problem.

The present study revealed important demographic features of firework-related injury. Firstly, boys under 10 years of age were the most susceptible population to firework-related eye injury. Consistent with this, Thygesen [19] reported that boys and young men are the main victims of firework-related eye injury. Similarly, a number of previous studies concluded that most firework-related eye injuries occur in children [7, 9, 20, 21]. There may be two main reasons for these results. Children have a poor sense of self-protection and may have accidents when guardians are negligent. Additionally, men may be more adventurous in trying dangerous things than are women. To avoid such tragedies, we need to strengthen early education for children, especially boys, and enhance parents' safety awareness and supervision of children. Secondly, most cases occurred during Spring Festival, which is consistent with previous reports. Jing et al. [22] reported on 24 firework-related eye injuries that occurred during Spring Festival, and Wang et al. [16] found that the peak time of firework injury was during Spring Festival. The reason for this result is obvious, as China traditionally lights fireworks during Spring Festival to bring good luck. Thus, additional protective measures must be enacted before Spring Festival, such as reinforcing the inspection of fireworks and handing out goggles. Thirdly, rural residents had a higher rate of firework-related eye injury than did urban residents, consistent with the report by Jing et al. [22]. The reasons for these results may be as follows: first, China imposed a ban on fireworks in most urban areas; second, there is a long history of household fireworks factories in rural areas [22]; third, a lower education level may lead to poor safety awareness in rural areas. Thus, actions such as accelerating the formulation of relevant laws, strengthening the safety inspection of household factories, and increasing the publicity of fireworks knowledge in rural areas should be undertaken.

The present study showed that the BCVA increased after treatment, relative to initial values, and the IOP tended to be within the normal range at the final visit. Similarly, Lin et al. [23] reported a statistically significant improvement in the BCVA after management for 53 eyes injured by fireworks. Additionally, Kong et al. [24] reported that the final BCVA in 118 eyes injured by fireworks reached a better level after treatment. Overall, surgical management was shown to be beneficial, even in patients with severe firework-related eye trauma. However, there are scarce data regarding the IOP in firework-related injury. The present study showed that undergoing the necessary surgery can restore the IOP to normal values, which is beneficial for functional recovery.

The top three injuries in the present study were cataract, RD, and CD, and the most frequent surgeries performed were cataract extraction, PPV, and PPL + PPV. These results differ from those of Kong et al. [24], in which the top three injuries were hyphema, vitreous hemorrhage, and corneal laceration. Furthermore, in Chang et al. [12], the most frequent injury types were corneal abrasion, hyphema, and eyelid injuries. The main reason for this difference in results may be as follows: the patients in the present study were exclusively inpatients, while previous studies included both inpatients and outpatients; as there is hierarchical medical system in China, patients might visit a basic medical institution to repair the open globe and visit a high-level hospital for advanced treatment. As different injuries lead to different surgeries, the most frequent surgeries in the present study were cataract extraction, PPV, and PPL + PPV, rather than corneal repair. It should be noted that 7% of patients in the present study underwent evisceration, which is similar to the rate of 5.9% in the study by Kong et al. [24]. This devastating medical outcome is a wake-up call, reinforcing the need for increased focus on this trauma.

The duration of hospitalization for firework-related eye injuries changed over time. Data regarding the length of the hospital stay for such injuries are rare. The present study showed that the number of hospitalization days decreased over time. Improvements in medical technology may be one reason for this change. However, another reason may be the implementation of day surgery management. Starting in 2015, the Zhongshan Ophthalmic Center began this mode of treatment, in which patients check in and out of the hospital within 24 hours [25]. In recent years, China has begun to implement day surgery. Zhuang et al. [26] reported that day surgery is cost-effective for patients with cataracts. To promote this diagnostic and treatment mode, the Zhongshan Ophthalmic Center has applied it to other eye diseases, including firework-related eye injury. The present study demonstrated that day surgery decreased the hospitalization time without adverse events, proving that this treatment mode is feasible for patients with firework-related eye injuries.

The main limitation of the present study is that we only utilized data from hospitalized patients who underwent surgeries at our center. Thus, outpatients whose injuries did not require surgery were not represented. As a result, the findings of the present study represent only serious firework-related eye injury cases and likely underestimate the true number of firework-related eye injuries in southern China. Larger studies, with more data regarding firework-related eye injury, are needed. In general, deeper research and greater effort are needed in this medical area.

## 5. Conclusions

The trend in firework-related eye injury was stable from 2013 to 2017, with a slight decline in 2016 and peaks during Spring Festival. Fireworks comprise the major cause of explosive eye injury, and boys under 10 years of age and rural residents are more susceptible to firework-related eye injury. The top three diagnoses in our study population were cataract, RD, and CD, and the major surgeries were cataract extraction, PPV, and PPL + PPV. The BCVA increased with treatment and the IOP tended to be normal at the final visit. Day surgery is a cost-effective treatment mode for patients with firework-related eye injury. Finally, we should raise awareness, strengthen legislation, and popularize relevant knowledge to better prevent this kind of ocular tragedy.

## Figures and Tables

**Table 1:** The initial and final IOP in patients with firework-related eye injury.

	Initial IOP		Final IOP	
	No. of eyes	%	No. of eyes	%
Normal	247	55.1	309	69^*∗*^
Abnormal	201	44.9	139	31

^*∗*^ *P* < 0.05, Chi-square test; normal: IOP within 10–21 mmHg/Tn; abnormal: IOP < 10 mmHg/Tn or >21 mmHg/Tn; IOP, intraocular pressure.

## Data Availability

The data used in this study are included in the article and are available upon reasonable request from the corresponding author.
